# Primary Colorectal Adenocarcinoma Metastatic to the Breast: Case Report and Review of Nineteen Cases

**DOI:** 10.1155/2011/738413

**Published:** 2011-06-22

**Authors:** Rodney E. Shackelford, Pushpa Allam-Nandyala, Marilyn M. Bui, John V. Kiluk, Nicole Nicosia Esposito

**Affiliations:** ^1^Department of Graduate Medical Education, H. Lee Moffitt Cancer Center & Research Institute, 12902 Magnolia Drive, Tampa, FL 33612, USA; ^2^Department of Anatomic Pathology, H. Lee Moffitt Cancer Center & Research Institute, 12902 Magnolia Drive, Tampa, FL 33612, USA; ^3^Department of Women's Oncology, Breast Program, H. Lee Moffitt Cancer Center & Research Institute, 12902 Magnolia Drive, Tampa, FL 33612, USA; ^4^Department of Pathology and Cell Biology, University of South Florida, Tampa, FL 33612, USA; ^5^Department of Oncologic Sciences, University of South Florida, Tampa, FL 33612, USA; ^6^Pathology & Laboratory Services (113), James A Haley VA Hospital, 13000 Bruce B Downs Boulevard, Tampa, FL 33612, USA

## Abstract

Metastases to the breast from extramammary primaries are uncommon and account for 0.5–6% of all breast malignancies (Georgiannos et al., 2001, and Vizcaíno et al., 2001). Malignant melanoma, lymphoma, and lung and gastric carcinomas are the most frequently encountered nonmammary metastases to the breast in adults (Georgiannos et al., 2001, and Chaignaud et al., 1994). Primary colorectal adenocarcinoma (CRC) metastatic to the breast is extremely rare, with the medical literature having only 19 recorded cases. Typically CRC metastatic to the breast is indicative of widely disseminated disease and a poor prognosis. Here we present a case of poorly differentiated colon cancer metastatic to the breast and review the current literature on this rare event.

## 1. Case Presentation

The following case was evaluated and reported in compliance with the University of South Florida's Institutional Review Board Policy no. 311.

**Figure 1:**
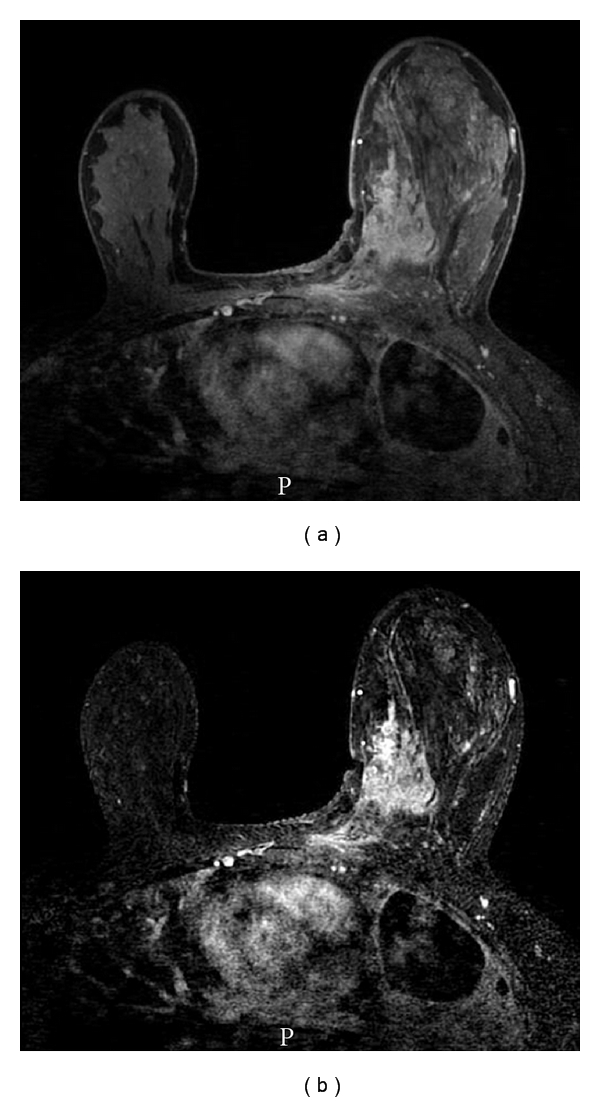
(a, b) Breast MRI demonstrated regional enhancement in the left superior breast extending to the chest wall and diffuse skin thickening.

**Figure 2:**
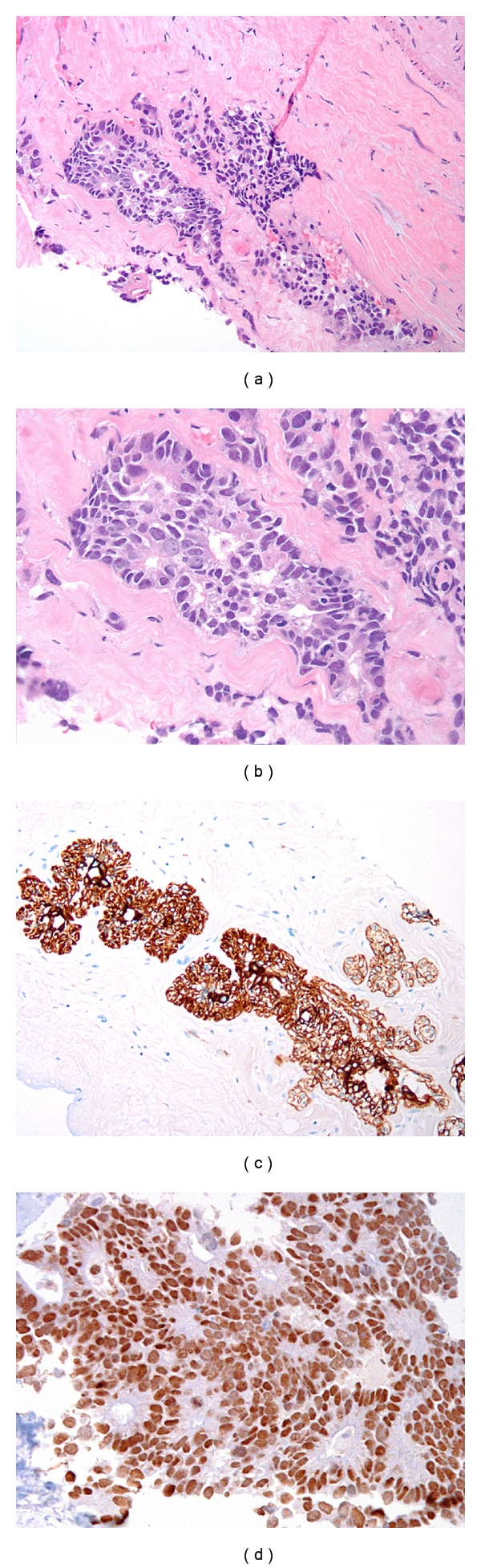
(a, b) The breast biopsy demonstrated an invasive adenocarcinoma with a focal cribriform pattern, hyperchromatic round to oval nuclei, and brisk mitotic activity (H&E, 100x, 200x). (c) Cytokeratin 20 showed diffuse, positive staining in the tumor (cytokeratin 20, 100x). (d) CDX2, specific for intestinal epithelium, exhibited diffuse nuclear immunoreactivity in the tumor cells (CDX2, 200x).

A 44-year-old Caucasian female was diagnosed with stage 1 (Duke's stage A) primary colonic adenocarcinoma in 2002 and treated with a colectomy. She did not receive adjuvant therapy, and was disease-free for seven years when she presented with nausea, vomiting, and poor coordination. CT scan revealed a left cerebellar mass, as well as multiple lung masses and mediastinal lymphadenopathy. The patient subsequently underwent resection of the cerebellar mass, which was diagnosed as metastatic CRC. The patient underwent palliative radiotherapy to the brain and chest and FOLFOX chemotherapy. After two chemoradiotherapy courses, she noticed a rapidly enlarging mass in her left breast. Clinical examination of the left breast revealed a mobile 11 × 8 cm mass in the upper outer quadrant. Breast ultrasound revealed a solid mass in the 9 and 10 o'clock regions of the left breast, and breast MRI demonstrated diffuse enhancement in the left medial superior breast which extended to the chest wall (Figure 1). The patient underwent core needle biopsy which revealed a poorly differentiated adenocarcinoma (Figure 2) with neither an in situ component nor lymphovascular space invasion. No tumor necrosis or cigar-shaped nuclei were identified to suggest a colonic primary. In light of the patient's clinical history, a panel of immunohistochemical stains was performed. The tumor was positive for CK20 and CDX-2 (Figure 2), and negative for CK-7, estrogen receptor, progesterone receptor, and HER2/neu by immunohistochemistry and fluorescent in situ hybridization (FISH). A diagnosis of metastatic colonic adenocarcinoma to the breast was thus rendered. 

## 2. Discussion

CRC metastatic to the breast is extremely rare, with the largest previous study reviewing only 8 cases [7]. Here we report a new case and review of 19 cases based on the current literature (Table 1). CRC with associated synchronous or metachronous breast metastases presents at an average age of 54.3 yrs with a range of 32–86 yrs, which is younger than the United States average of a primary CRC diagnosis at 72 yrs [18]. On average, breast metastasis is reported to appear 21 months following CRC diagnosis, with a range of 0 to 7 yrs, with only 3/20 (15%) cases first presenting as a breast lump, or as a breast lump with concurrent CRC. Three (15%) CRC breast metastases were in men, 15 (75%) in women, and no gender was reported in 2 (10%) cases. In 6 (30%) cases the patients died an average of 11 months after the diagnosis of breast metastasis, and the remaining 14 were either still alive at the time of the published case report or no survival data was reported. Fifteen (75%) cases had nonbreast metastases at the time of diagnosed breast metastasis, four reports did not mention other metastases, and in one case CRC breast metastasis was the only metastasis found. Metastases to the brain, liver, lung, skin, abdominal and axillary lymph nodes, and retroperitoneum were common. In one case, ocular and vertebral metastases were present [5]. The majority of metastases (55%) were to the left breast (often upper outer quadrant), with 6 (30%) to the right breast, and 2 bilateral (10%) [10]. Two of three reported male patients exhibited metastases in the left breast only [14, 15, 17]. CRC metastases to the breast averaged 3.5 cm in size and ranged from 1 to 11 cm. Most metastases were described as rapidly growing, discrete masses. CRC metastases to the breast most often appeared as masses without calcifications on mammography; in spite of the nine cases with reported mammographic findings, two (22%) exhibited microcalcifications (Table 2). Histopathology demonstrated an invasive adenocarcinoma, often with mucinous or signet-ring cell features, lacking an in situ component. Lymphovascular space invasion may be prominent. Finally, immunohistochemistry, when performed, revealed tumor cells to be positive for CDX-2, CK20, and CEA, and negative for CK7, ER, PR, HER2, and GCDFP-15, an immunophenotype typical of colorectal carcinoma [1–17]. 

Only a handful of CRC metastatic to the breast cases have been reported, with the two largest studies presenting two new cases each [13, 17]. After an extensive literature search, we located 19 cases. In light of the previously reported cases, the patient presented in this report exhibited some unusual aspects, having the largest breast metastasis (11 cm) and longest interval time from her initial CRC diagnosis to the diagnosed breast metastasis (7 years).

It is imperative to distinguish metastatic colorectal carcinoma to the breast from a primary mammary carcinoma, as the former requires systemic chemotherapy for colon cancer, while a primary breast cancer necessitates surgical management with or without breast-specific adjuvant therapy. Though histomorphologic clues of a metastatic CRC can be helpful, such as the presence of “dirty” necrosis, metastatic CRC can morphologically mimic a primary, poorly differentiated ductal carcinoma of the breast. Immunohistochemistry will in the vast majority of cases identify the phenotype of the carcinoma, as CRC is positive for cytokeratin 20 and CDX2, and negative for breast markers cytokeratin 7, GCDFP-15, mammaglobin, ER, and PR in greater than 90% of cases.

## 3. Conclusion

Combined with the case presented, CRC metastatic to the breast commonly (1) favors the left breast, (2) is accompanied by extensive metastatic disease to other organs, (3) exhibits rapid growth, (4) has a poor chemotherapy/radiation response, and (5) carries a poor prognosis secondary to disseminated disease. Radiologic studies of the breast may be misleading, as metastases may be associated with calcifications, and usually do not present as multiple, bilateral masses, thus mimicking a primary mammary carcinoma. Histopathologic clues of metastases include a lack of an in situ component, prominent lymphovascular space invasion, and a “triple-negative” phenotype. However, when histology offers no definitive clues of a metastasis, proper diagnosis of this rare event requires an accurate clinical history, proper immunohistochemical workup and high level of suspicion. This multidisciplinary approach is critical in avoiding unnecessary surgical procedures and pursuing proper subsequent patient management.

##  Conflict of Interests

The authors declared that there is no conflict of interests.

## Figures and Tables

**Table 1:** Colorectal carcinoma metastatic to the breast: summary of cases.

Reference	Sex	Age at CRC diagnosis	Primary colorectal carcinoma			Breast metastases		
			Interval between CRC and breast metastasis (years)	Location	Duke stage at presentation	Laterality	Location	Size(s) (cm)
Current report	F	39	7	Colon	A	Left	UOQ	11
[1]	F	68	1	Rectum	C1	Left	UOQ	n/a
[2]	F	42	0.5	Colon	n/a	Right	UQ	3
[3]	F	36	0.4	Rectum	n/a	Left	UOQ	6
[4]	F	77	0.25	Colon	C2	Left	UOQ	2
[5]	F	32	0.8	Rectum	n/a	Left	n/a	4
[6]	F	86	Concurrent	Colon	B	Right	UOQ	2
[7]	F	36	4	Colon	C	Left	UOQ	4
[8]	F	83	Concurrent	Colon	n/a	Left	n/a	n/a
[9]	F	48	5	Rectum	B	Left	LIQ	1
[10]	F	35	1	Colon	n/a	Bilateral	n/a	n/a
[11]	F	42	Concurrent	Colon	n/a	Right	UOQ/LOQ	n/a
[12]	F	43	Within weeks	Colon	D	Right	UOQ	4.5
[13]	F	43	2	Rectum	C	Bilateral	n/a	2 & 2.2
[13]	F	74	0.1	Colon	C	Bilateral	n/a	4 & 6
[14]	M	44	6	Colon	D	Right	n/a	1.5
[15]	M	66	1	Colon	B	Left	n/a	3
[16]	M	66	2.0	Colon	n/a	Left	Retroareolar	2.2
[17]	n/a	58.5	0.5	Colon	n/a	Left	n/a	3
[17]	n/a	66	2.0	Colon	n/a	Right	n/a	4

**Table 2:** Mammographic appearance of breast metastases.

Reference	Mammographic appearance
[2]	Mass
[4]	Mass
[7]	Ill-defined asymmetry with calcifications
[8]	Mass
[9]	Mass
[16]	Mass
[10]	Masses
[11]	Calcifications
[13]	Masses
